# A Novel Missense Mutation in Human Receptor Roundabout-1 *(ROBO1)* Gene Associated with Pituitary Stalk Interruption Syndrome

**DOI:** 10.4274/jcrpe.galenos.2019.2018.0309

**Published:** 2020-06-03

**Authors:** Ziqin Liu, Xiaobo Chen

**Affiliations:** 1 Capital Institute of Pediatrics, Clinic of Endocrinology, Beijing, China

**Keywords:** Receptor Roundabout-1 gene, pituitary stalk interruption syndrome, combined pituitary hormone deficiency, missense mutation

## Abstract

Pituitary stalk interruption syndrome (PSIS) is characterized by the association of an absent or thin pituitary stalk, an absent or hypoplastic anterior pituitary lobe and an ectopic posterior pituitary (EPP) lobe. The causes of this anatomical defect include both genetic and environmental factors. Molecular genetic defects have been indentified in a small number of patients with PSIS. A 4-year-old boy presented with hypoglycemia and hyponatremia associated with growth hormone, thyroid stimulating hormone, and adrenocorticotropic hormone deficiencies. The patient had right sided strabismus. magnetic resonance imaging images showed pituitary hypoplasia, EPP and absent pituitary stalk. A novel Receptor Roundabout-1 *(ROBO1)* missense mutation (c.1690C>T, p.Pro564Ser) that may contribute to the disorder was found in this patient and his mother, who also exhibited pituitary abnormalities.

**What is already known on this topic?**

Pituitary stalk interruption syndrome (PSIS) is a rare, congenital anomaly of the pituitary gland characterized by pituitary gland insufficiency, thin or discontinuous pituitary stalk, anterior pituitary hypoplasia, and ectopic positioning of the posterior pituitary gland. The underlying genetic etiology for the vast majority of cases remains to be determined.

**What this study adds?**

Heterozygous mutations of the human Receptor Roundabout-1 *(ROBO1)* gene were recently shown to be responsible for PSIS. Here, we used an exome sequencing approach to reveal a novel missense mutation (c.1690C>T, p.Pro564Ser) in *ROBO1* gene in a 4 year boy with with PSIS, The mutation was carried by his mother, whose pituitary magnetic resonance imaging showed also an abnormality.

## Introduction

Pituitary stalk interruption syndrome (PSIS) is a rare disorder due to the blocked transportation of hormones from the hypothalamus to the pituitary. The estimated incidence of this disorder is 0.5/100,000 births (1,2). Patients with PSIS are characterized by a combination of specific pituitary hormone deficiencies. There are typical findings evident on cranial magnetic resonance imaging (MRI), including interrupted or thin pituitary stalk, absent or ectopic posterior pituitary, and anterior pituitary hypoplasia (3,4). The diagnosis of this syndrome mostly depends on MRI imaging in combination with classical clinical and laboratory findings.

Despite intensive research into PSIS, the etiology remains unknown in 95% of cases, although genetic causes are suspected. In the human, mutations in *LHX4, OTX2, HESX1, SOX3, PROKR2, GPR161* and *CDON* have been postulated to be associated with PSIS. Recently, mutations in the Receptor Roundabout-1 *(ROBO1)* gene have been reported in five patients with PSIS (5), confirming its genetic association with PSIS.

Here, we report a case of PSIS with multiple anterior pituitary deficiencies and the classical triad of MRI findings, in which whole exome sequencing (WES) analysis identified a novel heterozygous mutation in the *ROBO1* gene. This mutation is also carried by his mother, who also had abnormal pituitary function and short stature, thereby providing strong circumstantial evidence for the association between this variant and the familial pituitary abnormalities.

## Case Report

**Figure 1:**
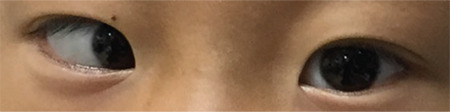
Image of the patient showing right strabismus

The patient was a 4-year-old boy who was admitted to our hospital with an episode of generalized tonic-clonic seizures. The episode was not associated with fever or any sign of infection. He was born fullterm at 39 weeks gestation and delivered with normal birth parameters (weight 2860 g: 10th-25th centile, length 50 cm: 50th centile). Psychomotor development was normal. At presentation the height of the patient was 97 cm (<3^th^ centile) and his weight was 16 kg (10^th^-25^th^ centile). Findings on physical examination were unremarkable apart from right strabismus (Figure 1). The penis was 2 cm (stretched) and the testicular volume was 1 mL bilaterally. The patient was the ﬁrst child of non-consanguineous Chinese parents. The father was healthy and of normal stature (170.5 cm, 25^th^-50^th^ centile), while the mother had a short stature (146 cm, <3^th^ centile). No further details of the mother’s medical history were available. His maternal grandmother also reported short stature and strabismus.

**Figure 2:**
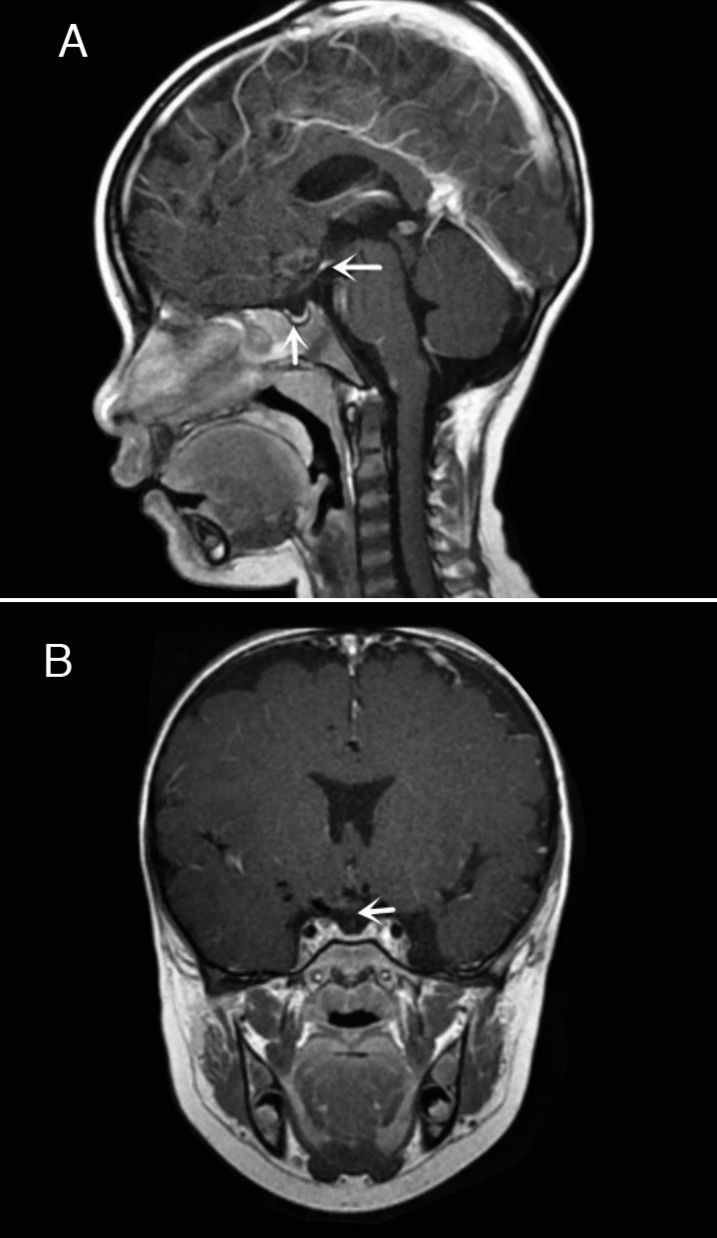
Sagittal and coronal magnetic resonance imaging of the pituitary confirming pituitary stalk interruption syndrome (A) Sagittal view. The small anterior pituitary (vertical arrow) and the posterior lobe was localized at the hypothalamic region (horizontal arrow). (B) Coronal view. The pituitary stalk is absent

The patient was found to have severe hypoglycemia with a blood glucose concentration of 0.92 mmol/L (normal, 3.3-5.5) and severe hyponatremia with a blood sodium (Na) of 117 mmol/L, (normal, 135-155). Hormone concentrations and biochemical parameters measured during hypoglycemia were as follows: serum insulin 0.9 mU/L, serum cortisol 0.55 ug/dL (normal, 6.2-19.4l), adrenocorticotropic hormone (ACTH) 9.8 pg/mL, urine ketone bodies were negative, plasma lactate 1.8 mmol/L (normal, <2) and serum ammonia 52.2 mol/L (normal, <80). The levels of creatinine kinase, creatine kinase MB fraction, organic acids, amino acids, acylcarnitines and free carnitine in plasma were normal. Blood gas analysis was pH 7.44, PCO_2_ 29.6 mmHg, HCO_3_ 22.4 mmol/L, BE -0.6 mmol/L. No abnormalities were detected on complete blood count. Hemoglobin A1c was 5.6% (normal, 4-6%). Growth hormone (GH) deﬁciency was attributed to this patient after insulin tolerance test and L-dopa test, with a peak GH of 0.3 and 0.05 ng/mL, respectively. Insulin like growth factor-1 (IGF-1) was 25 ng/mL (normal, 66-427) while prolactin was in the normal range. In addition to GH deficiency, he was diagnosed with central hypothyroidism [free T4, 8.6 pmol/L (normal, 10.8-20), thyroid stimulating hormone (TSH): 0.489 ulU/mL (normal, 0.8-5)]. Luteinizing hormone (LH) was 0.13 IU/L and follicle-stimulate hormone (FSH) 0.7 IU/L. The karyotype was 46,XY. Echocardiogram, electroencephalogram (EEG) and video-EEG showed no abnormalities. Cranial MRI revealed a small anterior pituitary gland, absent pituitary stalk and an ectopic posterior lobe (Figure 2). A diagnosis of PSIS was made based on these clinical and laboratory findings. The patient presented with combined pituitary hormone deficiency (CPHD) including GH deficiency, central hypothyroidism and central adrenocortical insufficiency. He was then treated with saline and hydrocortisone and a good response to this was obtained with stabilized blood sugar and blood Na concentrations. Subsequently, thyroxine (LT4) and GH replacement therapy were started.

## Genetic Analysis

DNA samples obtained from the family were sequenced to identify the causal gene using WES. DNA was isolated from peripheral blood using DNA Isolation Kit (Bioteke Corporation, AU1802, Wuxi, China). Genomic DNA samples (1 µg) were fragmented into 200-300 bp portions using a Covaris Acoustic System (Covaris, Woburn, MA, USA). The DNA fragments were then processed by end-repairing, A-tailing, adaptor ligation and a four-cycle pre-capture polymerase chain reaction amplification, after which all exons and the 50 bp bases in their adjacent introns were captured by SeqCap EZ Med Exome Enrichment Kit (Roche, Madison, WI, USA). Post-capture amplification and purification was performed on the DNA library and then sequenced on an Illumina HiSeq X Ten platform (Illumina, San Diego, CA, USA) mannually. The raw data produced were then filtered and aligned with the human genome reference (hg19) using the BWA Aligner (http://bio-bwa.sourceforge.net/) and variants were identified using NextGene V2.3.4 software (Soft genetics, LLC, State College PA, USA). The data had a 151.24× mean read depth and about 97.95% of the targetbases were covered at 20× average read depth.

The filtered variants were then annotated by using NextGene V2.3.4 and the laboratory’s own scripts to get related information, including the conservation of nucleotide bases and amino acids, prediction of the biological functions, frequency in normal populations (compared with 1000 Genomes, ExAC, dbSNP database and local specific databases), and the data from HGMD, Clinvar and OMIM. The potential effect of the variants were predicted by SIFT and Polyphen-2 (6,7,8). All variants of pathogenicity were interpreted according to the American College of Medical Genetics standards and categorized (9). The *ROBO1* gene has three transcripts recorded in the National Center for Biotechnology Information, of which NM_002941.3 was used as the reference sequence. Potentially pathogenic variants were verified using Sanger sequencing.

**Figure 3:**
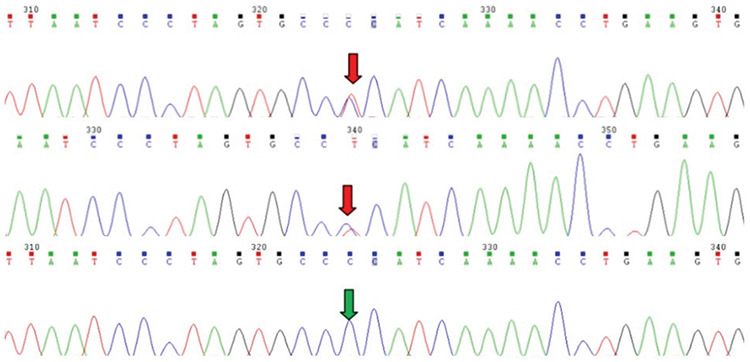
Sanger sequencing results of the family The heterozygous c.1690C>T, Pro564Ser *ROBO1* mutation is found in the patient (top) and his mother (middle). The father (bottom) has no mutation. The red arrows show the mutation

WES data filtering identified a heterozygous c.1690C>T, p.Pro564Ser variant (RefSeq: NM_002941.3; Chr3: 78717393) in the *ROBO1* gene (Figure 3). Segregation studies revealed that the mother was also a carrier of the same mutation. This rare sequence variant was further predicted to be “probably damaging” with a score of 0.999 in Polyphen-2 and “damaging” with a score of 0.01 by SIFT. Multiple amino acid sequence alignments showed that p.Pro 564 is highly conserved in human, Chimps (Pan troglodytes), mice (Mus musculus), zebra fish (Danio rerio), frogs (Xenopus tropicalis) and chickens (G gallus) (Table 1).

**Figure 4:**
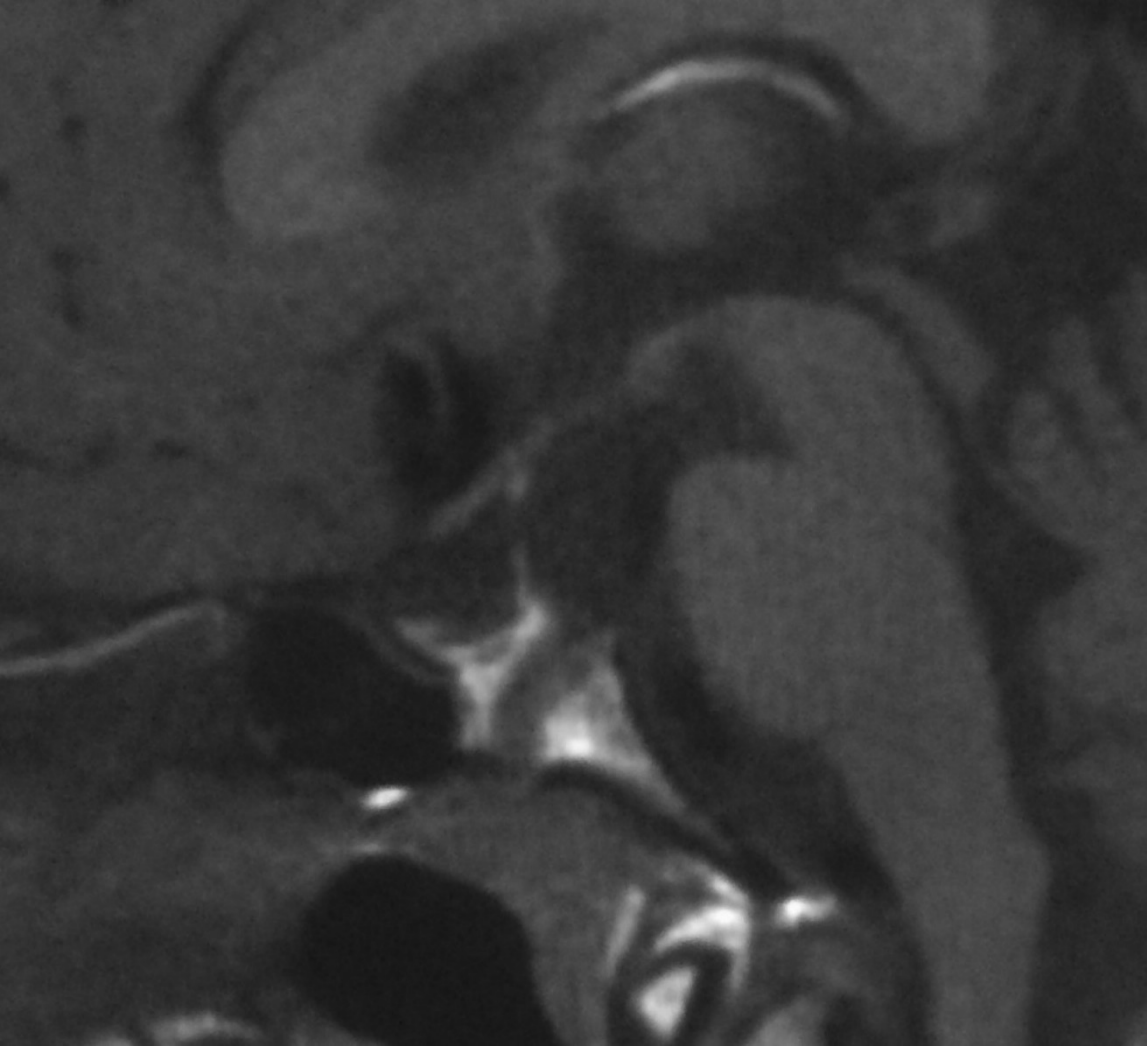
Magnetic resonance imaging (MRI) image from the patient’s mother. Her pituitary MRI showed a thin pituitary stalk and hypoplasia of the adenohypophysis

The mother, who has short stature, also carried the same *ROBO1* variant. She then underwent endocrine evaluation which showed an ACTH of 38.8 pg/mL, cortisol of 8.1 ug/dL, IGF-1 of 188 ng/mL (115-307 ng/mL), FT4 9.4 pmol/L, TSH: 2.16 ulU/mL, LH: 2.97 IU/L, FSH: 5.34 IU/L and E2 of 67 pg/mL. Her pituitary MRI showed a thin pituitary stalk with hypoplasia of the adenohypophysis (Figure 4).

## Discussion

The patient reported here had right strabismus and CPHD in GH, ACTH and TSH. His mother, who also carried the variant, had abnormal pituitary function, short stature but normal eye structure while the patient’s maternal grandmother had short stature and strabismus. Though DNA was not available from the deceased grandmother, this may reflect phenotypic variability in this family. The phenotypic variability found in the patient and his mother could be due to the impact of other genes in pituitary development or gene-environment interactions (10), and is similar reports of the missense variants involving *HESX1* and *LHX4* genes (11), of which the heterozygous variants are characterized by highly variable phenotypes amongst family members.

To date, several etiological factors have been proposed for PSIS, and there is good evidence for a polygenic etiology. *HESX1, LHX4, OTX2, SOX3*, and *PROKR2* have been reported to be associated with PSIS (12,13,14). In 2017, five unexplained PSIS cases including two famial cases identified one nonsense, one missense and one frameshift mutation (all heterozygous) in the *ROBO1* gene by WES (5) and this report was the first to identify novel heterozygous frameshift, nonsense and missense variants (p.Ala977Glnfs*40, two affected sibs; p.Tyr1114Ter, sporadic case and p.Cys240Ser affected child and paternal aunt) in *ROBO1* gene (Table 2) (15). Of these five cases, three showed isolated GH deficiency and the other two presented with combined GH and TSH deficiencies. Dateki et al (15) identiﬁed a novel homozygous splice site mutation in *ROBO1* (c.1342+1G>A) in a 5 year-old boy. The patient had CPHD, psychomotor developmental delay, severe intellectual disability, sensorineural hearing loss, strabismus, and characteristic facial features. Their findings suggest *ROBO1* gene as one of the potential causative genes of PSIS.The clinical phenotype of the patients harboring the *ROBO1* mutation varied in terms of the ocular and endocrine manifestations. In our report, by using next generation sequencing (NGS) technology, we identified a maternal missense mutation (c.1690C>T, p.Pro564Ser) in the *ROBO1* gene in a case diagnosed with PSIS and CPHD. This variant was predicted to be possibly pathogenic by Polyphen-2 and SIFT. Multiple amino acid sequence alignments showed that p.Pro 564 is highly conserved across various species including primates, other mammals birds and fish. All these findings suggest that this variant could play an important role in disease causation. Other patients who harbor *ROBO1* mutations have been reported to share some phenotypic features with the present patients. Four cases presented with strabismus and one case presented with ptosis. These data suggest that mutations in *ROBO1* contribute to ocular anomalies. Cardiomyopathy was seen in one patient and one patient had psychomotor developmental delay. More cases are needed to elucidate the relationship between genotype and phenotype. The *ROBO1* and its ligand Slit are known to influence axon guidance and central nervous system patterning in both vertebrate and nonvertebrate systems (16). Missing expression of *ROBO1* could lead to ectopic differentiation of forebrain neurons. The chemo repulsive ligand Slit and its receptors of the ROBO family are expressed in the developing and adult brain (17) and are crucially involved in the formation of midline commissures. Slit2 and Slit1/2 double knockout animals display defects in corticothalamic and thalamocortical targeting, callosal and hippocampal commissure projections (18) and defects in the formation of the optic chiasm. Calloni et al (19) described a 9-year-old boy with severe intellectual disability, absence of the transverse pontine fiber, thinning of the anterior commissure and corpus callosum, and compound heterozygous variants in the *ROBO1* gene. These findings strongly suggest that human *ROBO1* variants could result in neurodevelopmental disorders. Our patient was subsequently found to exhibit a wide range of symptoms, including classic CPHD and right strabismus. Thus the *ROBO1* gene may be one of the potential causative genes for PSIS and CPHD. Bjorke et al (20) showed that Slit signaling is necessary to inhibit the initiation of oculomotor neuron development. Oculomotor axons at the midline crossing are characterized by an axon-like process that forms from the cell body as a secondary axon. It may be possible that this repolarization is subject to ROBO regulation. Overall, the introduction of NGS technology in the diagnostic workflow will lead to the identification of novel genetic determinants in pediatric patients with pituitary defects (21,22). There is mounting evidence that ROBO variants are associated with ocular as well as pituitary abnormalities.Additional examples of *ROBO1* variants and clinical PSIS cases are needed to explore the function of *ROBO1* and its effect on human embryogenesis and organogenesis.

## Figures and Tables

**Table 1:**
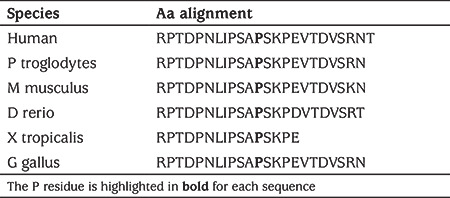
Alignment of amino acid sequences encoded by the *ROBO1* gene from different species

**Table 2:**
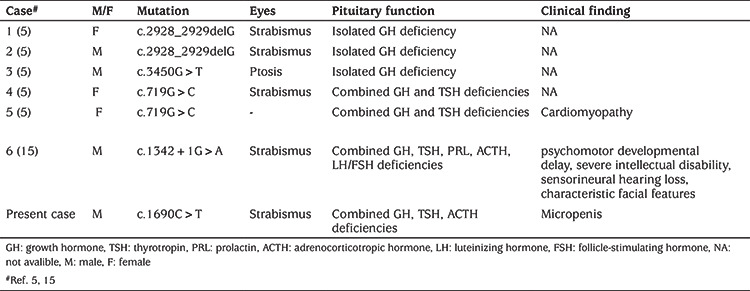
Clinical and genetic features of patients with *ROBO1* mutations in pituitary stalk interruption syndrome
